# A pilot study: the relationship between salivary MCP-1 and IgA, and exercise performance in long-distance runners and sprinters

**DOI:** 10.1186/s13104-022-05989-2

**Published:** 2022-03-26

**Authors:** Masataka Uchida, Tadashi Suga, Masafumi Terada, Tadao Isaka

**Affiliations:** 1 Ritsumeikan-Global Innovation Research Organization, Ritsumeikan University, 1-1-1 Nojihigashi, Kusatsu, Shiga 525-8577 Japan; 2 Research Organization of Science and Technology, Ritsumeikan University, Kusatsu, Shiga Japan; 3 Faculty of Sport and Health Science, Ritsumeikan University, Kusatsu, Shiga Japan

## Abstract

**Objective:**

It remains unclear that the relationship between sprint and/or endurance performance and salivary immunological factors and stress hormones in athletes. The aim of this study was to investigate if salivary immunological factors and stress hormones are related to sprint and endurance performance in sprinters and long-distance runners. Fourteen male sprinters provided 100-m record and 22 male long-distance runners provided 5000-m record. Salivary IgA, MCP-1, interleukin-8, and cortisol levels in sprinters and long-distance runners were measured by ELISA assay.

**Results:**

No significant differences were found in all salivary parameters between sprinters and long-distance runners. In long-distance runners, the salivary IgA and MCP-1 concentrations and secretory rate significantly correlated with their personal best 5000-m times (r = 0.534, P = 0.011; r = 0.567, P = 0.006; r = 0.452, P = 0.035, respectively). In sprinters, the salivary IgA concentration, MCP-1 concentration, and MCP-1 secretory rate did not correlate with personal best 100-m sprint times (r = − 0.260, P = 0.369; r = 0.128, P = 0.663; r = 0.122, P = 0.677, respectively). Therefore, the present study is the first to determine that immunological factors such as IgA and MCP1 may be related to endurance performance in long-distance runners.

## Introduction

A biomarker is a biological substance contained in a biological sample (e.g., blood, urine, and saliva) and is an objective indication of biological and pathological process. Previous researchers have utilized biomarkers as key parameters for assessing physical and physiological fitness, conditioning, overtraining, stress status, fatigue, and performance in athletes [1]. To assess an individual's performance and health among athletes, a single biomarker cannot be used alone, and a combination of multiple biomarkers must be considered. Furthermore, it is important to identify the biomarkers that match different characteristics of various sports [1].

Associations of conditioning, which is related to exercise performance, with stress hormones and immunological factors has been well studied. Circulating cortisol levels are considered stress and fatigue indicators in response to exercise intensity and duration [2]. Cortisol is a potential biomarker for overtraining syndrome, which is characterized by a reduction in sport-specific physical performance, excessive fatigue, and subjective symptoms of stress [3]. Cortisol is induced by prolonged exercise and required for adaptation of exercise, such as protein and lipid catabolism, to increase gluconeogenesis and reduce inflammation. Immunoglobulin A (IgA) is the main immunological factor in external secretions for protective effects against pathogen infections. Salivary IgA production is reduced by strenuous exercise [4]. Salivary IgA levels progressively decrease in swimmers during periods of intensified training [5]. Previous study reported that serum interleukin-8 (IL-8) levels after the race are strongly and negatively correlated with the time taken to finish the marathon in marathon runners presenting with exercise-induced bronchoconstriction [6]. A previous study has suggested that the amount of change in salivary monocyte chemotactic protein 1 (MCP-1) and proinflammatory markers (e.g., IL-8) following high-intensity sprint tasks may be associated with lower drop jump and squat jump performance [7]. Investigators of the previous study speculated that the secretion of MCP-1 from damaged skeletal muscles in response to exercise may significantly contribute to reductions in muscular function. Therefore, each of the aforementioned markers may predict performance in athlete.

Saliva sampling is a useful method for the assessment of stress hormones and immunological parameters in response to exercise [8]. Compared to blood sampling, which is typical method for assessment of stress hormones and immunological parameters, salivary sampling is a convenient noninvasive method suitable for continuous collection [9, 10]. Salivary biomarkers have been proven as useful tools in sports science research [11]. Previous study showed that salivary IgA levels were inversely correlated with the greater number of infection cases in swimmers, and salivary IgA levels during a competitive season could predict the number of infection cases in the elite swimmers [5]. While the relationship between salivary biomarkers and physical conditioning in athletes has been well studied [11], the relationship between salivary biomarkers and exercise performance in athletes is unclear.

In track and field, performance for sprinting and long-distance running is associated with multiple factors, such as morphological and genetic factors, muscle functional parameters, and muscle mechanical properties [12–15]. However, the contribution of saliva immunological factors and stress hormone to athletic performance in competitive sprinters and endurance runners is currently unknown. Therefore, this study aimed to determine if salivary immunological factors and stress hormones are related to sprinting and endurance performance in collegiate sprinters and long-distance runners. The physiological factors, which interact as determinants of athletic performance, are typically different between sprinters and endurance runners. Thus, we hypothesized that the associations of athletic performance with saliva immunological factors and stress hormone would differ between sprinters and long-distance runners.

## Main text

### Methods

Fourteen well-trained male sprinters and twenty-two well-trained male long-distance runners participated in this study. Sprinters’ personal best 100-m sprint times ranged from 10.37 to 11.78 s (11.13 ± 0.36 s). Long-distance runners’ personal best 5000-m times ranged from 843 to 936 s (880.40 ± 27.61 s). All participants were informed of the experimental procedures and provided written consent to participate in the study. This study was approved by the Ethics Committee of Ritsumeikan University (BKC-IRB-2017-009).

All athletes were instructed not to eat or drink fluids other than water for at least 12 h prior to the measurement. The athletes came to the laboratory without breakfast. Before collection of saliva samples, heights and body mass were measured. These measurements were taken during pre-season. Participants were asked to consume 250 ml or more of water after waking up.

Saliva samples were collected between 8:00 and 10:00 a.m. Athletes completely rinsed their mouths with distilled water (for 30 s, three times) and then rested for at least 10 min, and saliva production was stimulated by chewing a sterile sponge (Salivette; Sarstedt Inc., Germany) at a frequency of 60 times per minute. Saliva samples were centrifuged (3000 rpm, 15 min, 4 °C). After measurement of the sample volume, saliva samples were stored at − 80 °C until enzyme-linked immunosorbent assays (ELISA) were performed.

Salivary S-IgA (Bethyl, MN, USA), cortisol (SALIMETRICS, CA, USA), MCP-1, and IL-8 (R&D Systems, MN, USA) concentrations were measured using a commercial ELISA kit. Saliva samples were centrifuged for 1 min at 10,000 rpm prior to measurement. Salivary parameters secretory rates were calculated by multiplying each salivary parameter’s absolute concentration by the saliva flow rate (ml/min).

Data are presented as mean ± standard deviation. A separate unpaired t-test was used to compare dependent variables between sprinters and long-distance runners. The relationship between salivary parameters and personal best 100-m sprint time and/or personal best 5000-m time in sprinters and long-distance runners were examined using Pearson’s product-moment correlation coefficient. The statistical significance level was set at p < 0.05. All statistical analyses were conducted using IBM SPSS software (version 27.0; International Business Machines Corp., USA).

### Results

The physical characteristics and salivary parameters of the sprinters and long-distance runners are listed in Table 1. Body height, body mass, and body mass index were significantly higher in sprinters than in long-distance runners.

**Table 1:** Physical characteristics and salivary parameter

	Sprinter (n = 14)	Long-distance runner (n = 22)
Age	20.8 ± 0.8	20.5 ± 0.1
Body height, cm	177.47 ± 5.57	171.15 ± 4.36*
Body weight, kg	68.40 ± 5.10	58.10 ± 4.42*
BMI,	21.70 ± 1.22	19.81 ± 0.96*
Salivary volume, ml	1.48 ± 0.44	1.08 ± 0.26*
Cortisol, mg/dl	0.14 ± 0.08	0.13 ± 0.07
IgA, mg/ml	93.44 ± 43.69	98.20 ± 50.56
MCP-1, pg/ml	345.02 ± 341.15	334.10 ± 284.03
IL-8, pg/ml	87.07 ± 96.71	145.26 ± 182.48
Cortisol secretory rate, ng/min	2.29 ± 1.84	1.43 ± 0.73
IgA secretory rate, mg/min	134.49 ± 64.22	105.65 ± 55.33
MCP1 secretory rate, pg/min	516.87 ± 502.42	354.05 ± 293.39
IL8 secretory rate, pg/min	87.07 ± 96.72	145.26 ± 182.48

Values are means ± standard deviation. IgA: immunoglobulin A; MCP-1: monocyte chemotactic protein 1; IL-8: interleukin-8; BMI: body mass index

*p < 0.05 vs. sprinter

Salivary volume was significantly higher in sprinters than in long-distance runners. None of the salivary parameters significantly differed between sprinters and long-distance runners.

**Fig. 1:**
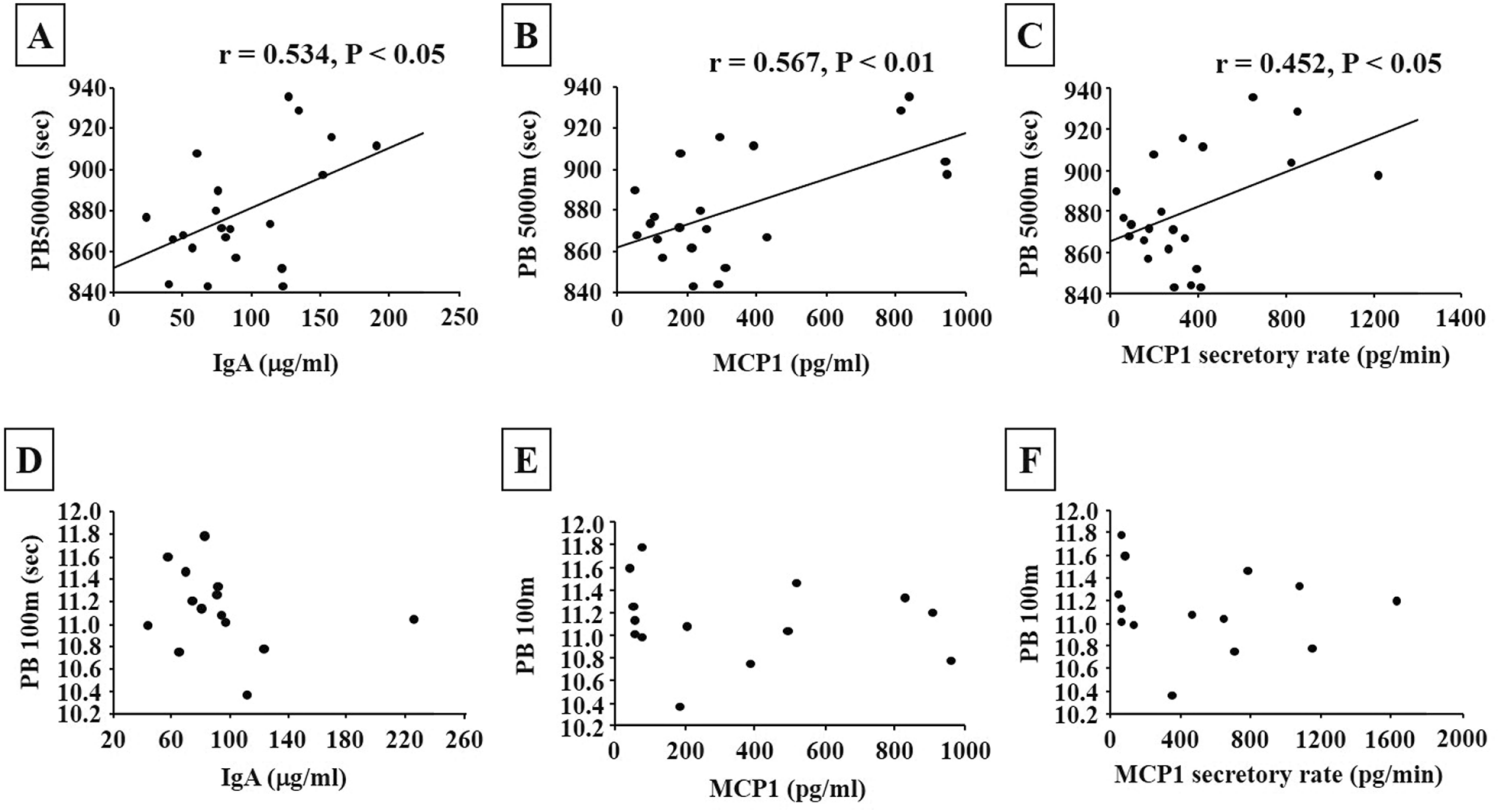
Correlation between performance outcomes and salivary parameters in long-distance runners (**A**–**C**) and sprinters (**D**–**F**). **A**, **C** Show correlation between performance outcomes and salivary IgA concentration. **B**, **E** Show correlation between performance outcomes and salivary MCP-1 concentration. **C**, **F** Show correlation between performance outcomes and salivary MCP-1 secretory rate

The salivary S-IgA concentration in long-distance runners significantly correlated with their personal best 5000-m times, with a positive correlation (r = 0.534, P = 0.011; Fig. 1), but not salivary S-IgA secretory rate (r = 0.324, P = 0.141). Moreover, the salivary MCP-1 concentration and secretory rate in long-distance runners significantly correlated with their personal best 5000-m times, with a positive correlation (MCP-1 concentration: r = 0.567, P = 0.006; MCP-1 secretory rate: r = 0.452, P = 0.035; Fig. 1).

The salivary S-IgA concentration, MCP-1 concentration, and MCP-1 secretory rate in sprinters did not correlate with their personal best 100-m sprint times (S-IgA concentration: r = -0.260, P = 0.369; MCP-1 concentration: r = 0.128, P = 0.663; MCP-1 secretory rate: r = 0.122, P = 0.677; Fig. 1). Furthermore, Salivary cortisol levels and IL-8 levels in long-distance runners did not correlate with their personal best 5000-m times (cortisol concentration: r = 0.163, P = 0.469; cortisol secretory rate: r = − 0.074, P = 0.743; IL-8 concentration: r = 0.290, P = 0.190; IL-8 secretory rate: r = 0.229, P = 0.304), but not personal best 100-m sprint times (cortisol concentration: r = − 0.212, P = 0.468; cortisol secretory rate: r = − 0.251, P = 0.387; IL-8 concentration: r = − 0.384, P = 0.175; IL-8 secretory rate: r = − 0.481, P = 0.082) in sprinters.

### Discussion

In this study, we demonstrated that salivary immunological parameters and stress hormones did not differ significantly between sprinters and long-distance runners. We further demonstrated that salivary IgA concentration, salivary MCP-1 concentration, and secretory rate positively correlated with personal best 5000-m times in long-distance runners. In sprinters, salivary immunological parameters and stress hormones were not correlated with sprint performance. Therefore, these findings suggest that salivary IgA and MCP-1 levels might be associated with endurance performance in long-distance runners.

To the best of our knowledge, this firs study to report salivary IgA concentration positively correlated with the personal best 5000-m times in long-distance runners. Previous research observed reductions in salivary IgA concentration after ultramarathon race in well-trained runners [16], while another investigation reported that changes in salivary IgA concentration did not contribute to running distances during a game in football players [17]. Our results of salivary IgA levels and endurance performance would seem to contradict the findings of the previous study [17]. A potential explanation of differences in results between their work and the current study may be the differences in characteristics of sport. The associations between sprint performance and salivary IgA levels have not been previously investigated. This is the first study to identify the potential associations between sprint performance and salivary IgA levels. This study indicate salivary IgA concentration may contribute to personal best 100-m times in sprinters. Previous investigation has reported that salivary IgA concentration is transiently reduced by moderate intensity aerobic exercise [18]. However, high intensity interval exercise does not reduce salivary IgA concentration [19]. It is possible that the difference in the responsiveness of salivary IgA levels to exercise is related to the difference in the contribution of salivary IgA to performance between long-distance runners and sprinters. While the present study provided novel data to confirm the associations between salivary IgA levels and athletic performance, further research is needed to explore effects of difference in the　characteristics of the sport on salivary IgA levels.

In this study, salivary MCP-1 concentration and secretory rate were associated with endurance performances, such as positive correlation with personal best 5000-m times, in long-distance runners. Previous study has reported that circulating MCP-1 concentrations were increased by aerobic moderate-intensity training [20]. Additionally, end-systolic wall stress (ESWS) negatively correlated with peak oxygen uptake in adult subjects with systemic right ventricles [21], plasma MCP-1 levels positively correlated with ESWS in adult subjects with dilated cardiomyopathy [22]. MCP-1 level may be involved in cardiac function associated with VO_2_, potentially indicating that MCP-1 level may contribute to endurance performance. There are no studies that have observed the relationship between sprint performance and MCP-1. In this study, there was no relationship between sprint performance and salivary MCP-1 levels in sprinters. Whereas, previous study has reported that circulating MCP-1 concentrations did not change after high-intensity interval training or sprint intensity training [20, 23]. Findings from these previous studies and our results indicate that changes in basal salivary MCP-1 levels may reflect endurance performance in long-distance runners, although more studies are needed to better understand the role of MCP-1 in exercise performance in long-distance runners.

In conclusion, the present study demonstrated that lower levels of basal salivary IgA and MCP-1 were related to higher endurance performance in long-distance runners. To the best of our knowledge, this pilot study provides evidence that, in addition to physiological and morphological factors, immunological factors such as salivary IgA and MCP-1 may play an important role in the superior endurance performance of long-distance runners. From a clinical perspective, information from the present study may help in recognizing individual conditions and predicting physical performance in athletes. In addition, our findings indicate that the relationship between salivary immunological biomarkers and physical performance may be influenced by differences in characteristics of sports in track and field athletes. Furthermore, as these biomarkers have potentials to influence performance, they could be utilized to identify individual fatigue, stress, and health status, which may be useful in developing match strategies and practice plans. Therefore, information from the present study can be further utilized in basic knowledge of biomarkers for optimal training/conditioning programs and for improving outcomes in long-distance runners.

### Limitation

Because the salivary IgA and MCP-1 levels did not differ between sprinters and long-distance runners, we could not provide clear data to support our hypothesis that athletes with higher endurance performance have lower salivary IgA and MCP-1 levels. To test our hypotheses, further studies are needed to examine the relationship between endurance performance and salivary immunological biomarkers in other sports. Additionally, sprint performance-related immunological markers and stress hormones in saliva were not found in sprinters. To assess sprint performance-related salivary biomarkers in sprinters, further studies are needed. Because we included only collegiate sprinters and endurance runners in this study, the observed associations may not be applicable to other athletic populations. Further research is needed for the non-athlete and other sports athlete. Lastly, we cannot eliminate potential effects of different training cycles, competitive levels or competitive seasons on the observed associations between selected salivary markers and athletic performance. In the future, it is necessary to examine the contribution of the salivary markers to athletic performance during a competition season and training cycles.

## Data Availability

Data will be provided the corresponding author (M.U.) upon request.

## Abbreviations

IgA: Immunoglobulin A
MCP-1: Monocyte chemotactic protein 1
IL-8: Interleukin-8
